# Ironing out Persisters? Revisiting the Iron Chelation Strategy to Target Planktonic Bacterial Persisters Harboured in Carbapenem-Resistant *Escherichia coli*

**DOI:** 10.3390/microorganisms12050972

**Published:** 2024-05-12

**Authors:** Jia Hao Yeo, Nasren Begam, Wan Ting Leow, Jia Xuan Goh, Yang Zhong, Yiying Cai, Andrea Lay-Hoon Kwa

**Affiliations:** 1 Department of Pharmacy, Singapore General Hospital, Singapore 169608, Singapore; yeo.jia.hao@sgh.com.sg (J.H.Y.);; 2 SingHealth-Duke-NUS Academic Clinical Programme (Pathology), Singapore 169857, Singapore; 3 Department of Clinical Translational Research, Singapore General Hospital, Singapore 169856, Singapore; 4 SingHealth-Duke-NUS Academic Clinical Programme (Medicine), Singapore 169857, Singapore; 5 Emerging Infectious Diseases, Duke-National University of Singapore Medical School, Singapore 169857, Singapore

**Keywords:** antibiotic persistence, carbapenem-resistant *Escherichia coli*, time-kill study, iron chelation

## Abstract

Antibiotic resistance is a global health crisis. Notably, carbapenem-resistant Enterobacterales (CRE) pose a significant clinical challenge due to the limited effective treatment options. This problem is exacerbated by persisters that develop upon antibiotic exposure. Bacteria persisters can tolerate high antibiotic doses and can cause recalcitrant infections, potentially developing further antibiotic resistance. Iron is a critical micronutrient for survival. We aimed to evaluate the utility of iron chelators, alone and in combination with antibiotics, in managing persisters. We hypothesized that iron chelators eradicate CRE persisters in vitro, when administered in combination with antibiotics. Our screening revealed three clinical isolates with bacteria persisters that resuscitated upon antibiotic removal. These isolates were treated with both meropenem and an iron chelator (deferoxamine mesylate, deferiprone or dexrazoxane) over 24 h. Against our hypothesis, bacteria persisters survived and resuscitated upon withdrawing both the antibiotic and iron chelator. Pursuing our aim, we next hypothesized that iron chelation is feasible as a post-antibiotic treatment in managing and suppressing persisters’ resuscitation. We exposed bacteria persisters to an iron chelator without antibiotics. Flow cytometric assessments revealed that iron chelators are inconsistent in suppressing persister resuscitation. Collectively, these results suggest that the iron chelation strategy may not be useful as an antibiotic adjunct to target planktonic bacteria persisters.

## 1. Introduction

Antibiotic resistance is a global health crisis. *Escherichia coli* (under the order of Enterobacterales) is predicted to be the current top contributor to antibiotic resistance worldwide [1]. Notably, carbapenem-resistant Enterobacterales (CRE) pose a significant clinical challenge due to the limited effective treatment options. In Singapore, clinical CRE has been trending among adult inpatients since 2010 [2,3,4]. As numerous antibiotics are becoming ineffective, the World Health Organization is encouraging the search for novel therapeutic options and treatment strategies against ESKAPE organisms. Encountering a dying pipeline for the development of new antimicrobials, many attempts have been made to re-purpose existing drugs that were intended to treat other diseases.

Iron plays critical roles in many physiological functions, such as energy production [5] and regulating metabolism [6]. Hence, removing or chelating iron compromises microbial survival. This could be a feasible anti-microbial solution to the drying antimicrobial developmental pipeline. Classic iron chelators, such as deferoxamine mesylate (DFO) and deferiprone (DFP), are FDA-approved iron chelators that have used in clinics for decades to treat anemia and iron-overload disorders [7,8]. Dexrazoxane (DXZ) is a licensed drug that treats cardiomyopathy associated with doxorubicin, used to treat metastatic breast cancer, by chelating iron [9,10]. Other novel effective iron chelator compounds, such as 2,2-bipyridyl (commonly known as DIBI), are not currently approved for use in clinics. Hence, the assessments of these iron chelators are less useful for immediate translational applications.

Recent evidence demonstrates that a combination of both an iron chelator and antibiotics eradicates biofilms and potentiates the action of antibiotics [11,12,13,14,15,16,17]. Furthermore, iron chelation was shown to alleviate inflammatory symptoms triggered by the immune system upon exposure to bacteria [18,19,20]. In this study, we revisited the iron chelation strategy as a therapeutic option.

Bacteria persisters are a small subpopulation of bacteria that are dormant, non-dividing and survive antibiotic challenges. The emergence of bacterial persisters is a major concern that contributes to the development of pan-drug-resistant bacterial infection, for which no treatment is effective [21]. It is unknown if a combination of an iron chelator and an antibiotic would have any effect against bacteria persisters.

Hence, the aim of this study is to evaluate the utility of iron chelators, both alone and in combination with antibiotics used in eradicating and managing bacterial persisters. Given the importance of iron for organism survival, we hypothesized that a combination of both antibiotics and an iron chelator could eradicate persisters completely. Following our primary aim, we will also proceed to evaluate the utility of iron chelation as a post-antibiotic treatment option in managing and suppressing the resuscitation of persisters.

## 2. Materials and Methods

### 2.1. Bacteria

Nonclonal clinical strains of CR *E. coli* were previously collected as part of a nationwide surveillance study from 2011 to 2012 and were obtained from the largest tertiary hospital in Singapore (1700 beds). Genus identities were determined using Vitek 2 ID-GN cards (bioMérieux, Inc., Hazelwood, MO, USA). CR *E. coli* strains were stored at −80 °C in Cryobank (Thermo Scientific, Singapore) storage vials. Fresh isolates were sub-cultured twice on 5% sheep blood agar plates (Thermo Scientific, Singapore) for 24 h at 35 °C before each experiment.

### 2.2. Flow Cytometry

#### 2.2.1. Fluorochromes Used for Flow Cytometry

Bacteria were labelled with 150 µM CFSE (Thermo Fisher Scientific, Singapore) prior to antibiotic exposure. CFSE is a cell division marker that forms stable conjugates by binding irreversibly to aliphatic amines [22]. Upon cell division, the CFSE fluorescence is halved, enabling individual cell division events in the population to be identified [22]. This dye has previously been shown to be non-toxic to bacteria, even at high concentrations [23,24].

To further differentiate between viable and non-viable bacteria, we employed a combination of propidium iodide (PI) and SYTO-62 dyes (Thermo Fisher Scientific, Singapore). SYTO-62 labels the nucleic acid of all bacteria, while PI only enters and intercalates into the DNA of non-viable cells with compromised membranes.

#### 2.2.2. Flow Cytometric Data Acquisition and Data Analysis

A Cytoflex^®^ flow cytometer (Beckman Coulter, Brea, CA, USA) with the basic 4 + 3 + 2 configuration was used in this study. Flow cytometric data were acquired using the complementing CytExpert software (version 2.5). A flow rate of 50 to 150 events per second was used to acquire the samples. A total of 10,000 SYTO-62^POSTIVE^ events were acquired per sample. Further details on flow cytometer configurations and sample acquisition settings are detailed in the Supplementary Information.

Acquired flow cytometric data were analysed using FlowJo software (version 10.6; Treestar^®^ FlowJo, LLC, Ashland, OR, USA). Compensation against spectral overlap was also performed on the FlowJo software (version 10.6; Treestar^®^ FlowJo, LLC, Ashland, OR, USA) prior to data analyses. The gating strategy used for data analyses is shown in the Supplementary Information.

### 2.3. Time-Kill Studies (TKS)

#### 2.3.1. Time-Kill Assessments via Viable Plating

Time-kill studies (TKS) were performed on all 12 strains with antibiotics and respective concentrations are listed in Table 1. Antibiotic concentrations were based on the maximal clinically relevant unbound concentrations when maximum antibiotic doses were administered (see individual references in Table 1). Procedures for the TKS are described in detail in our previous studies [23]. Briefly, 15 mL of log-phase bacterial suspensions in Ca-MHB was transferred to sterile flasks containing 1 mL of antibiotics (and an iron chelator) and placed into an incubator maintained at 35 °C. The final inoculum concentration was approximately 5 log_10_ CFU/mL. At specific time intervals (0, 0.5, 1, 1.5, 2, 4, 24 h), samples were aliquoted in duplicate from each flask. Viable counts were obtained by depositing serial 10-fold dilutions of the reconstituted samples onto Mueller–Hinton agar (MHA) plates (Thermo Scientific, Singapore). Plates were incubated at 35 °C for 16–20 h. The colonies that formed were enumerated at 24 h. The lower limit of detection for the colony counts was determined to be 2.6 log_10_ CFU/mL.

#### 2.3.2. Time-Kill Assessments Using Flow Cytometry

Bacteria cultures were grown overnight in Ca-MHB at 35 °C until log phase was reached. The log-phase bacterial culture was then diluted in Ca-MHB to an inoculum of approximately 6.6 log_10_ CFU/mL. One milliliter of diluted bacterial culture was washed twice with phosphate-buffered saline (PBS) before staining with 150 μM CFSE in 0.02% (v/v) Triton X-100 (Sigma-Aldrich, Singapore) for 30 min in a shaking incubator at 30 °C [31]. Excess CFSE was then quenched with cold 10% (v/v) fetal bovine serum (FBS) (Thermo Fisher Scientific, Singapore) in PBS, centrifuged at 3000 rpm at 4 °C for 30 min, and resuspended in Ca-MHB. Bacterial suspensions in Ca-MHB were then transferred to sterile flasks containing 16 mL of antibiotics with/without an iron chelator and placed into a shaking incubator maintained at 35 °C. The final inoculum was approximately 5 log_10_ CFU/mL.

At specific time intervals (0, 0.5, 1, 1.5, 2, 4, 24 h), 1 mL of samples were aliquoted from each flask. CFSE-labelled bacteria were further stained with 20 μM propidium iodide and 1 µM SYTO-62 for 15 min prior to flow cytometric assessments.

Stained bacteria were then assessed using a Cytoflex flow cytometer (Beckman Coulter, Brea, CA, USA). A steady flow rate of approximately 150 events/s was acquired per sample. A total of 10,000 SYTO-62^POSITIVE^ events were collected for each sample. The acquired data were analyzed on the FlowJo software version 10.6.1 (FlowJo, LLC, Ashland, OR, USA). The gating strategy used to analyze data is presented in Supplementary Figure S4. Further details can be found in the Supplementary Information.

### 2.4. Determining Minimum Inhibitory Concentrations (MIC) and Minimum Bactericidal Concentrations (MBC)

Both MIC and MBC were performed on both the parent and resuscitated bacterial strains. Standard broth dilution method was used to determine the MIC of meropenem for the isolates. All assessments were performed following the CLSI guidelines.

## 3. Results

### 3.1. A Combination of Clinically Approved Iron Chelators and Meropenem Did Not Eradicate Bacteria Persisters

Previously, our flow cytometric approach identified persisters in carbapenem-resistant *Acinetobacter baumannii* clinical isolates upon exposure to both polymyxin B and rifampicin simultaneously [23]. We screened 12 carbapenem-resistant *Escherichia coli* clinical isolates using five different antibiotics. Amongst these sixty bacteria-to-antibiotic combinations, three isolates (EC0210, EC0238, EC0381) were identified to have fulfilled our criteria as manifesting as persisters when exposed to meropenem (Supplementary Figures S1–S3, Supplementary Tables S1–S3). These clinical strains were isolated from various infected sites, harboured different sets of resistant genes and had different serotypes (Supplementary Table S2).

Having identified the drug-manifested persisters in the clinical isolates, we tested our first hypothesis that a combination of both iron chelators and antibiotics can eradicate persisters completely.

**Figure 1:**
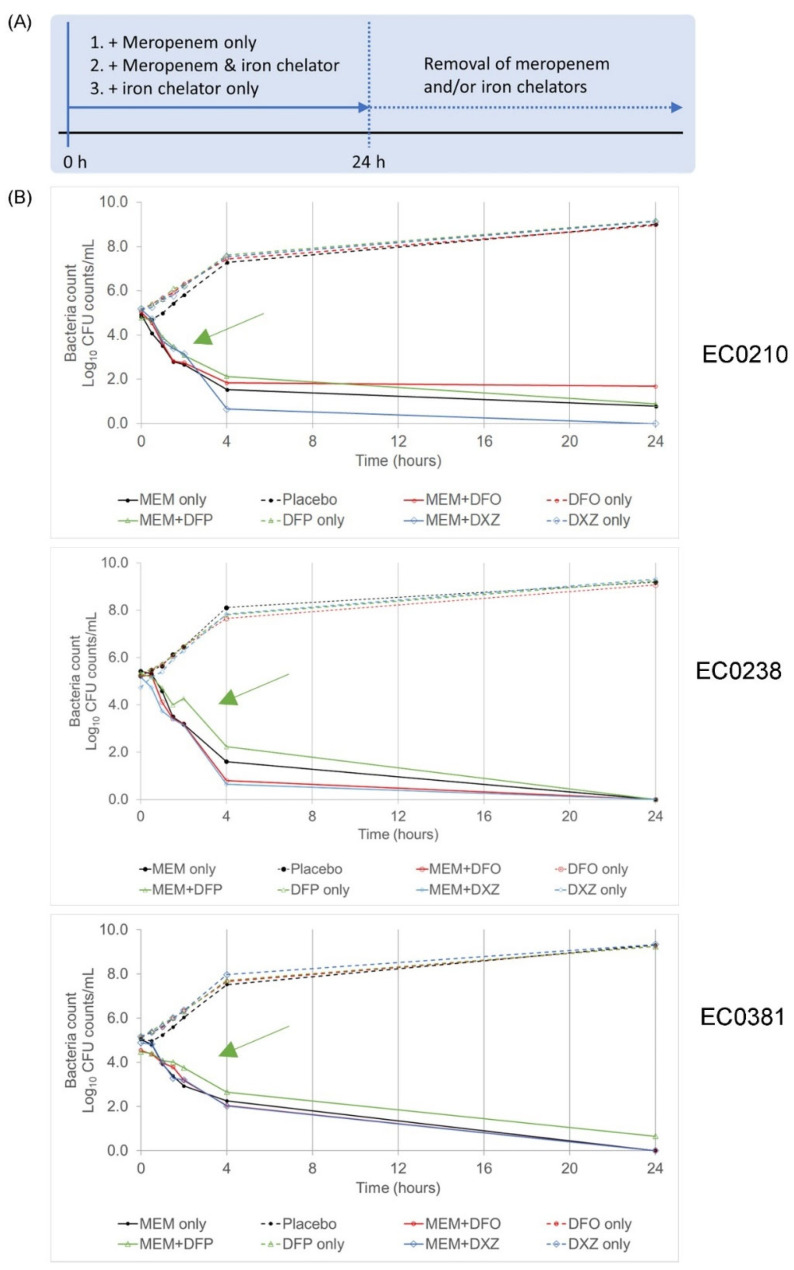
A combination of meropenem (MEM) and deferiprone (DFP) revealed slower bactericidal killing dynamics in comparison to other treatments. (**A**) Experimental design of the combination of both meropenem and iron chelator to eradicate bacteria persisters. (**B**) Graphs showing colony, enumerated from viable plating for respective clinical isolates. Green arrow in each graph points to the respective plot showing colonies enumerated from a treatment combination of meropenem+deferiprone (MEM + DFP).

Combinations of both meropenem (MEM) and an iron chelator (deferiprone (DFP), deferoxamine mesylate (DFO) or dexrazoxane (DXZ)) were simultaneously added to the three isolates identified earlier (EC0210, EC0238, EC0381). Clinically achievable concentrations of the drugs were used (Table 1). Time kill studies were conducted, assessing the bactericidal event over 24 h using viable plating and flow cytometric analyses (Figure 1A). The iron levels in culture media were estimated to be 0.8 mg/L ([32]). Both DFO and DXZ chelate iron were observed at a ratio of 1:1 (iron–iron chelator), whereas DFP chelates iron was observed at a ratio of 1:1 (iron–iron chelator). Therefore, the concentrations of iron chelators used in this study exceeded the iron levels within the culture media by more than 10 times. Hence, the influence of metal ions in the culture media and the formation of chelate complexes is negligible.

Colonies enumerated from viable plating revealed identical bactericidal effects for meropenem+DFO and meropenem+DXZ combinations when compared to meropenem alone in all three isolates (Figure 1B). On the other hand, all isolates treated with meropenem+DFP combinations revealed slower killing dynamics compared to meropenem alone, as shown by the higher bacterial counts obtained at the same timepoints (Figure 1B, green arrows).

**Figure 2:**
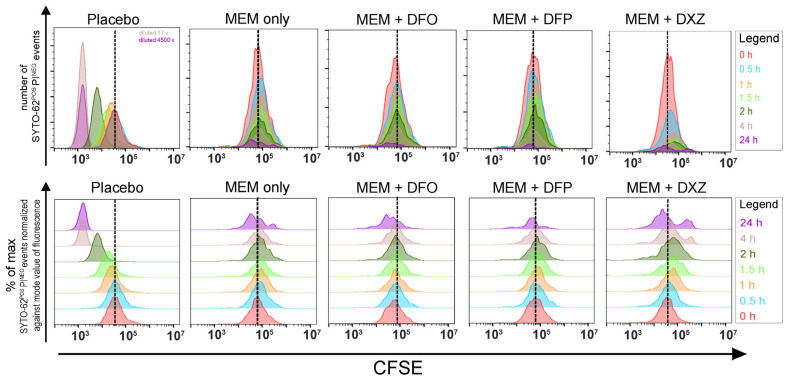
A sub-population of bacteria survived meropenem and all combinations of meropenem with an iron chelator. Representative flow cytometric plots demonstrating a small sub-population of bacteria from the EC0381 strain that survived meropenem and all combinations of meropenem with an iron chelator. Top panel shows overlaid histograms to demonstrate increasing or decreasing viable bacteria (SYTO-62^POSITIVE^PI^NEGATIVE^ events) across treatments. Bottom panel shows staggered histograms to visualize the shifts in fluorescence intensities over time. The dotted lines mark the fluorescence intensity peak of histogram at 0 h. Colors of histograms correspond to the timepoints, as stated in the legend.

As a bacteria cell divides, CFSE fluorescence is halved. Flow cytometric analyses revealed diminishing CFSE fluorescence and increasing viable bacteria counts (SYTO-62^POSITIVE^PI^NEGATIVE^ events) over time in the placebo controls. When exposed to meropenem along or meropenem with an iron chelator, the isolates exhibited decreasing bacteria counts. These decreasing counts concurred with the decreasing colony counts from viable plating that were revealed over time. A sub-population (identified as CFSE^HIGH^SYTO-62^POSITIVE^PI^NEGATIVE^ phenotype) was observed at 24 h in both exposure to meropenem alone and all meropenem–iron chelator combinations (Figure 2). This suggests that bacteria persisters were not eradicated in the meropenem–iron chelator combination.

To validate the flow cytometric observations of bacteria persisters surviving the 24 h drug treatment, these bacteria survivors were resuscitated in fresh broth. For all strains, bacteria could be resuscitated in all iron chelator–meropenem combinations, as indicated by the turbidity of fresh broth (Table 2). The similar morphologies observed in the TSA blood agar further validate the persisters’ resuscitation. Against our hypothesis, this evidence collectively indicates that an iron chelator–antibiotic combination could not fully eradicate bacterial persisters.

### 3.2. Iron Chelators Are Inconsistent in Suppressing Persister Resuscitation in All Strains

**Figure 3:**
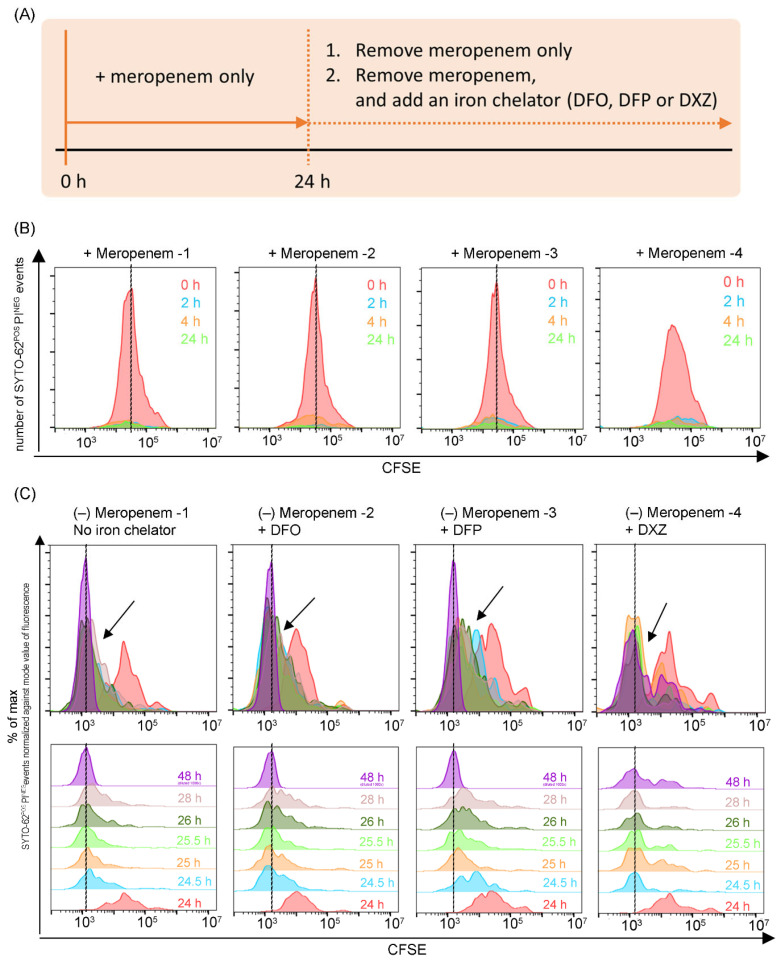
Flow cytometric analyses reveal deferiprone (DFP) delays the resuscitation of bacterial persisters in the EC0210 strain. (**A**) Experimental design for determining if iron chelation would suppress the resuscitation of bacteria persisters. (**B**) Meropenem treatment reduces the number of viable bacteria. Dotted lines refer to baseline at 0 h. (**C**) Flow cytometric analyses of persisters after the removal of meropenem with or without addition of iron chelator for each condition. Black arrows point to delayed resuscitation by DFP. Dotted lines refer to baseline at 48 h.

Following our aim, we proceeded to evaluate the utility of iron chelation as a post-antibiotic treatment option in managing and suppressing the resuscitation of persisters. Isolates were first treated with meropenem for 24 h. Exposure to meropenem led to identical bactericidal events during our screening process. Meropenem was then removed rapidly via multiple washing steps. Pelleted bacterial survivors were then treated with clinical achievable concentrations of an iron chelator (DFO, DFP or DXZ), and were assessed by flow cytometry during persister resuscitation (Figure 3A,B).

For all the bacterial strains (EC0210, EC0238 and EC0381), flow cytometric analyses showed an immediate diminished CFSE fluorescence right after DFO or DXZ was added, similar to the no-iron-chelator control. Representative data from clinical isolate EC0210 are presented in Figure 3C. This indicates that the bacteria actively divide after the removal of meropenem. Against our hypothesis, neither DFO nor DXZ suppresses resuscitation.

When bacteria persisters from both EC0238 and EC0381 isolates were exposed to DFP, flow cytometric analyses revealed immediate diminishing CFSE fluorescence. However, a momentary delay in bacterial growth was observed for the EC0210 isolate when DFP was added after removing meropenem. This was indicated by the slower diminishing of CFSE fluorescence after 24 h. However, DFP did not completely suppress the resuscitation of the bacterial persister from the EC0210 strain (Figure 3C, black arrows; Table 3). This is postulated to be due to the different virulence and resistance mechanisms in each isolate. Collectively, this evidence demonstrates that iron chelators are incapable of consistently suppressing persister resuscitation in all strains.

## 4. Discussion

Antibiotic resistance is a global health crisis. Notably, carbapenem-resistant Enterobacterales (CRE) pose a significant clinical challenge due to the limited effective treatment options. *E. coli* is the predicted to be the current top contributor to antibiotic resistance worldwide [1]. The presence of CRE bacterial persisters exacerbates the AMR problem, which contributes to the development of the pan-drug-resistant CRE, for which no treatment can be effective. The dying pipeline for the development of new antimicrobials has resulted in attempts to re-purpose existing drugs intended for treating other diseases as antimicrobials.

Iron is a micronutrient that stimulates bacterial growth, and is essential for diverse biological functions, such as DNA replication/repair, gene expression regulation, redox buffering (Fe-S clusters), oxygen hosts (porphyrins and heme) and glucose metabolism (such as aconitases). In a bacterial infection, hosts limit the survival and proliferation of microbial pathogens, using iron-binding proteins to hide iron away, while pathogens develop devious strategies against hosts to scavenge iron. Iron chelation therapy has been used to treat bacterial infections for many decades. However, the use of iron chelators alone has failed miserably when they are used as antibiotics, with MICs of >512 µg/mL in bacteria reference strains [33].

In recent years, iron chelation has repeatedly been demonstrated to be effective in destabilizing biofilms and potentiating antibiotics’ actions in numerous organisms [11,12,13,14,15,16,17]. These new insights have rekindled interest in iron chelation strategies as a therapeutic option against bacterial infections. Biofilms are responsible for over 60% of all bacterial infections in developed countries [34]. Biofilms were calculated to harbor a high number of bacteria persisters [35]. Hence, we expected that a similar antibiotic potentiation to be observed in planktonic persisters, as biofilm-encased persisters are more tolerant of antibiotics than planktonic persisters. Instead, our study demonstrated that the use of iron chelators as adjuncts did not eradicate persisters, contradicting the effectiveness of iron chelation in targeting biofilms. This strongly suggests that iron may play a different planktonic role in persisters, as opposed to being encased in biofilms.

DFO has long been used as an iron chelator in clinics. Consistent with other studies, DFO was concluded to not be a suitable candidate as an antimicrobial—alone or in combination with other drugs. Furthermore, DFO can also act as a siderophore for iron uptake in other bacteria species. Similar to DFO, deferasirox is also widely used in clinics for treating chronic iron overload diseases. However, we omitted the use of deferasirox due to solubility issues. DXZ is a licensed drug that prevents cardiomyopathies associated with anthracycline toxicities, typically doxorubicin [10]. DXZ is also a catalytic inhibitor of DNA topoisomerase II [36]. Structurally, DXZ is a bisdioxopiperazine that readily enters cells and is subsequently hydrolyzed as a chelator analogous to EDTA [37]. This chelating property is proposed to be its mechanism of action in the prevention of anthracycline-induced, iron-dependent, free-radical oxidative stress on the cardiac muscle [9]. To the best of our knowledge, this is the first time DXZ is compared to bacteria isolates as an iron chelator. Similar to the results regarding DFO, we conclude that DXZ is not a feasible candidate for use as antibiotic adjunct.

In this study, the use of DFP with antibiotics led to a slower bactericidal trend, and the use of DFP alone delayed bacterial persister resuscitation. DFP is a small molecule, with a molecular weight of 139.2 g/mol. We reasoned that our observations were due to the small DFP penetrating the bacteria and directly chelating intracellular iron. This contrasts the results obtained for both DFO and DXZ, which are bigger molecules than DFP, and hence, creating an iron-limiting environment that could compromise bacteria survival.

Iron is a double-edged sword. Iron is a critical micronutrient for survival and is involved in processes such as bacterial growth and metabolism. However, excess iron undergoes Fenton chemistry, generating reactive oxygen species (ROS) that radicalize biomolecules in cells, resulting in bacterial cell death. Evidence suggests that antibiotic exposure alters bacterial iron homeostasis and, subsequently, leads to large amounts of ROS accumulating in bacteria [38,39,40,41]. The removal of iron reduces the amount of ROS that is accumulated, and hence, reduces cell death. This may explain the phenomenon that DFP delays the bactericidal event in all isolates. Due to the lack of a good fluorescent probe to quantify intracellular bacterial iron using flow cytometry, it is difficult to assess the extent of iron chelation by DFP in each single bacterium.

However, our reasoning of Fenton-chemistry-induced bactericidal effects creates a new paradox. This paradox asks if iron supplements should be given, instead of iron chelators, as antibiotics adjuncts to treat bacterial infections. A seemingly plausible solution to answer this paradox is, perhaps, a specific delivery to bacteria only, containing both iron and antibiotics. This delivery system is similar to that of cefiderocol, which is deemed the Trojan Horse. However, both oral and intravenous iron supplements have been shown to have detrimental effects in infected hosts [42,43,44]. Furthermore, there are increasing reports on resistance against cefiderocol in multiple ESKAPE bacteria species that are resistant to carbapenems [45,46,47,48,49]. Hence, we express our curiosity regarding iron metabolism and trafficking in bacteria persisters.

There are challenges in this study. As these bacteria persisters are present in very low numbers, one technical challenge is accurately quantifying persisters. These drug-induced persisters’ numbers are below the limit of detection (10^4^) on our flow cytometer [23], which is similar to the limit of detection reported using other flow cytometers [50,51,52,53,54]. The increase in bacteria persisters under iron-limiting conditions may still be well below our limit of detection. As discussed in our previous work [23], persisters cannot be enumerated using traditional viable plating methods. This is further substantiated in this work, with no colonies forming on MH agar at the 24 h timepoint, while bacteria persisters were still observed by fluorescence microscopy. Therefore, it is not possible to conclude if iron-limiting conditions increase the number of eliminating bacterial persisters using conventional flow cytometry.

In conclusion, clinically approved iron chelators at clinically achievable concentrations do not serve as good candidate antibiotic adjuncts to planktonically target bacteria persisters. Our future studies will attempt to evaluate other candidates in our aim to eliminate bacteria persisters.

## Figures and Tables

**Table 1:** List of drugs and respective concentrations used in this study.

Antibiotics (Abbreviations)		Company	Final Concentration (mg/L)	Reference
Amikacin		TRC ^1^	65	Tod et al., 1998 [25]
Ceftazidime–avibactam (CZA)	Ceftazidime	TRC ^1^	21	Stein et al., 2019 [26]
	Avibactam	Pfizer	5.25	
Deferiprone (DFP) ^2^		ChemScene	26.49	Bellanti et al., 2014 [8]
Deferoxamine mesylate (DFO) ^2^		ChemScene	43.7	Ratha et al., 2013 [7]
Dexrazoxane (DXZ) ^2^		ApexBio	36.5	Jirkovský et al., 2018 [27]
Levofloxacin (LVX)		Daiichi	8	Rebuck et al., 2002 [28]
Meropenem (MEM)		TRC ^1^	20	Tam et al., 2005 [29]
Polymyxin B (PMB)		TRC ^1^	2	Kwa et al., 2008 [30]

Iron chelators and antibiotics were prepared in sterile ultrapure MilliQ water.^1^ TRC: Toronto Research Centre; ^2^ Concentrations of these iron chelators determined were established as peak plasma concentrations in respective references.

**Table 2:** Bacteria colony enumeration and resuscitation after antibiotic and/or drug treatment.

Conditions		EC0210	EC0238	EC0381
No antibiotic	Log_10_ CFU/mL at 24 h	9.03	9.24	9.36
Meropenem (MEM) only	Log_10_ CFU/mL at 24 h	1.69	0	0
	Resuscitate after removal of MEM?	Yes	Yes	Yes
MEM + Deferoxamine mesylate (DFO)	Log_10_ CFU/mL at 24 h	1.69	0	0
	Resuscitate after removal of MEM + DFO?	Yes	Yes	Yes
MEM + Deferiprone (DFP)	Log_10_ CFU/mL at 24 h	0.89	0.95	0.65
	Resuscitate after removal of MEM + DFP?	Yes	Yes	Yes
MEM + Dexrazoxane (DXZ)	Log_10_ CFU/mL at 24 h	0	0	0
	Resuscitate after removal of MEM + DXZ?	Yes	Yes	Yes

**Table 3:** Iron chelators were inconsistent in suppressing resuscitation.

	Resuscitate at 48 h in the Presence of Iron Chelators?		
	EC0210	EC0238	EC0381
Deferoxamine mesylate (DFO)	Yes	No	Yes
Deferiprone (DFP)	Yes	No	Yes
Dexrazoxane (DXZ)	No	Yes	No

## Data Availability

Data are contained within the article.

## Supplementary Materials

The following supporting information can be downloaded at: https://www.mdpi.com/article/10.3390/microorganisms12050972/s1, Figure S1: Bacteria persisters manifest in clinical isolate EC0210 after meropenem exposure; Figure S2: Bacteria persisters manifest in clinical isolate EC0238 after meropenem exposure; Figure S3: Bacteria persisters manifest in clinical isolate EC0238 after meropenem exposure; Figure S4: Gating strategy for flow cytometry to identify persisters; Table S1: Minimum inhibitory concentrations (MIC) and minimum bactericidal concentrations (MBC) measured for each parent and resuscitated strains post-antibiotic treatment; Table S2: Resistance gene and molecular information of the clinical isolates; Table S3: MICs of respective antibiotics screened for clinical isolates identified to manifest with persisters; Table S4: Flow cytometry acquisition summary; Table S5: Acquisition settings on the Nikon Ti-Microscope. References [55,56] are cited in the Supplementary Materials.
