# Supplementary material for: Ironing out Persisters? Revisiting the Iron Chelation Strategy to Target Planktonic Bacterial Persisters Harboured in Carbapenem-Resistant Escherichia coli
Source: Microorganisms. 2024 May 12;12(5):972. doi: 10.3390/microorganisms12050972 (PMC11123761; doi:10.3390/microorganisms12050972)
Supplement: Supplementary file 1 [file microorganisms-12-00972-s001.zip › Yeo et al_Ironing out the persisters (Sup)_Microorganism.pdf]

Supplementary Information contents

1. Results

- A. Screening carbapenem resistant clinical isolates for persisters
- B. Details of clinical isolates manifested with persisters

2. Materials and methods

- A. Flow cytometry
  - I. Flow cytometer configuration and settings
  - II. Sample acquisition settings
  - III. Data analyses
- B. Widefield epi-fluorescence microscopy of live bacteria
  - I. Staining live bacteria for fluorescence microscopy
  - II. Fluorescence microscope configuration and image acquisition

3. References (for supplementary information)

1. Results

A. Bacteria persisters manifest in carbapenem-resistant *Escherichia coli* clinical isolates when exposed to meropenem

In this study, we adopted a flow cytometric protocol to identify carbapenem-resistant *Escherichia coli* persisters in clinical isolates. Previously, our flow cytometric approach identified persisters manifested in carbapenem-resistant *Acinetobacter baumannii* clinical isolates, upon exposure to both polymyxin B and rifampicin simultaneously (1).

A total of twelve *E. coli* clinical isolates was screened for drug-induced persisters against various antibiotic treatments. These antibiotics include amikacin, ceftazidime-avibactam, levofloxacin, meropenem and polymyxin B. The antibiotics concentrations determined were concentrations achievable clinically (**Table 3**).

To identify persisters with good confidence in this study, we established a set of criteria based on the consensus established made by multiple leading research groups (2). The criteria established for identifying persisters are as follows:

(1) Observable bactericidal effect rather than growth inhibition by the antibiotic;

(2) Bacteria exposed to the antibiotic treatment reveal a bi-phasic bactericidal curve over a duration of 24 hours;

(3) Bacteria persisters retained high carboxyfluorescein succindyl-ester fluorescence ($CFSE^{HIGH}$) observable by fluorometric methodologies such as flow cytometry.

(4) Bacteria persisters that survives antibiotic treatment are able to resuscitate upon removal of antibiotic (3)

(5) Minimum concentration of antibiotic to inhibit growth (i.e., minimum inhibitory concentrations, MIC) is similar between resuscitated cells and parent strain.

We assessed a total of 60 bacteria-to-antibiotic combinations, screening 12 isolates over 5 different antibiotics. Amongst these 60 bacteria-to-antibiotic combinations, three isolates (EC0210, EC0238, EC0381) were identified to have fulfilled our criteria to manifest as persisters when exposed to meropenem. Results obtained from the screening process for EC0210, EC0238, and EC0381 were shown in **Supplementary Figure S1**, **Supplementary Figure S2** and **Supplementary Figure S3** respectively. The MICs against each antibiotic screened and the resistance gene details of these isolates can be found in **Supplementary Table S2** and **Supplementary Table S3** respectively.

A variety of techniques were employed to screen for bacterial persisters induced by antibiotics. Time-kill studies using both flow cytometry and viable plating showed bactericidal effects by meropenem (Criterion 1). All 3 isolates exhibit a bi-phasic bactericidal curve over 24 hours as observed with traditional viable plating (Criterion 2). Flow cytometric analyses revealed viable bacteria that survived meropenem treatment retained high carboxyfluorescein succindyl-ester ($CFSE^{HIGH}$) fluorescence in all 3 isolates (Criterion 3). These $CFSE^{HIGH}$ bacteria survivors were visually validated using fluorescence microscopy. These surviving bacteria in all 3 isolates could be resuscitated upon removal of meropenem (Criterion 4). The minimum inhibitory concentrations of meropenem, determined using broth microdilution methods, were similar between resuscitated cells and parent strain (Criterion 5, **Supplementary Table S1**).

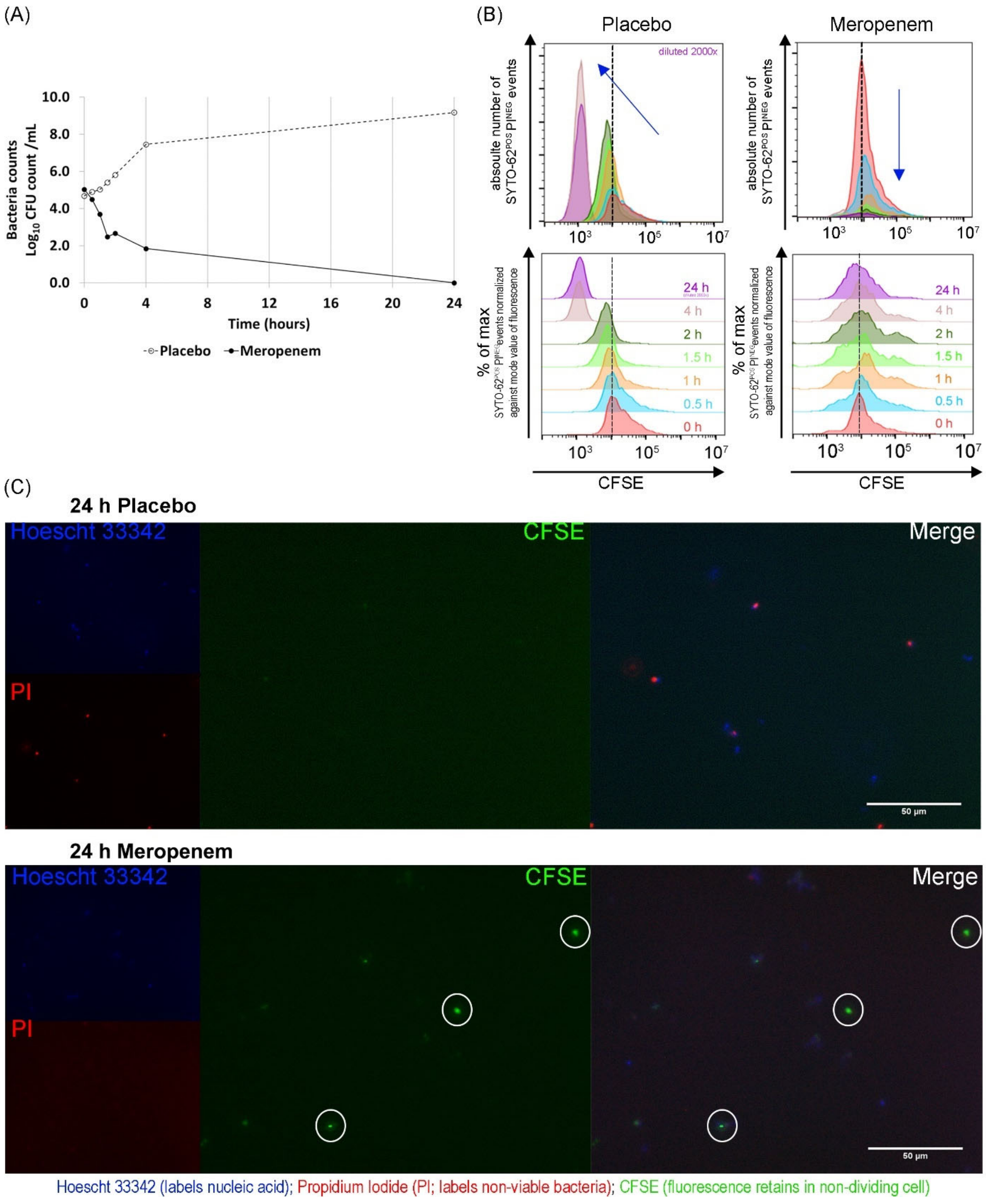

Supplementary Figure S1: Bacteria persisters manifest in clinical isolate, EC0210, after meropenem exposure.

This figure presents representative data from clinical isolate (EC0210) exhibiting presence of persisters after exposing to meropenem. (A) Graphs showing colonies enumerated exhibiting a bi-phasic curve upon meropenem treatment, in comparison to placebo. (B) Flow cytometric histograms revealing a sub-population of bacteria

surviving meropenem treatment. Dotted line marked the peak fluorescence intensity at 0 hour. Blue arrow on placebo overlaid histogram plot indicates the increasing viable bacteria events and leftward shift of CFSE fluorescence intensities over time. Blue arrow on meropenem treated histogram plots indicates decreasing viable bacteria and no change in CFSE fluorescence intensities over time. (C) Widefield epi-fluorescence microscopy revealing presence of $CFSE^{High}$ bacteria, noted in white circles, when treated with meropenem at 24 hours. Scale bar represents 50 microns.

Table S1: Minimum inhibitory concentrations (MIC) and minimum bactericidal concentrations (MBC) measured for each parent and resuscitated strains post-antibiotic treatment.

***E. coli* strains**	**Meropenem**	**Parent strain**	**Resuscitated strain**
EC0210	MIC[1]	4 mg/L	8 mg/L
	MBC[2]	16 mg/L	32 mg/L
EC0238	MIC	8 mg/L	8 mg/L
	MBC	16 mg/L	128 mg/L
EC0381	MIC	8 mg/L	8 mg/L
	MBC	32 mg/L	32 mg/L

[1] MIC: Minimum Inhibitory Concentration; [2] MBC: Minimum Bactericidal Concentration

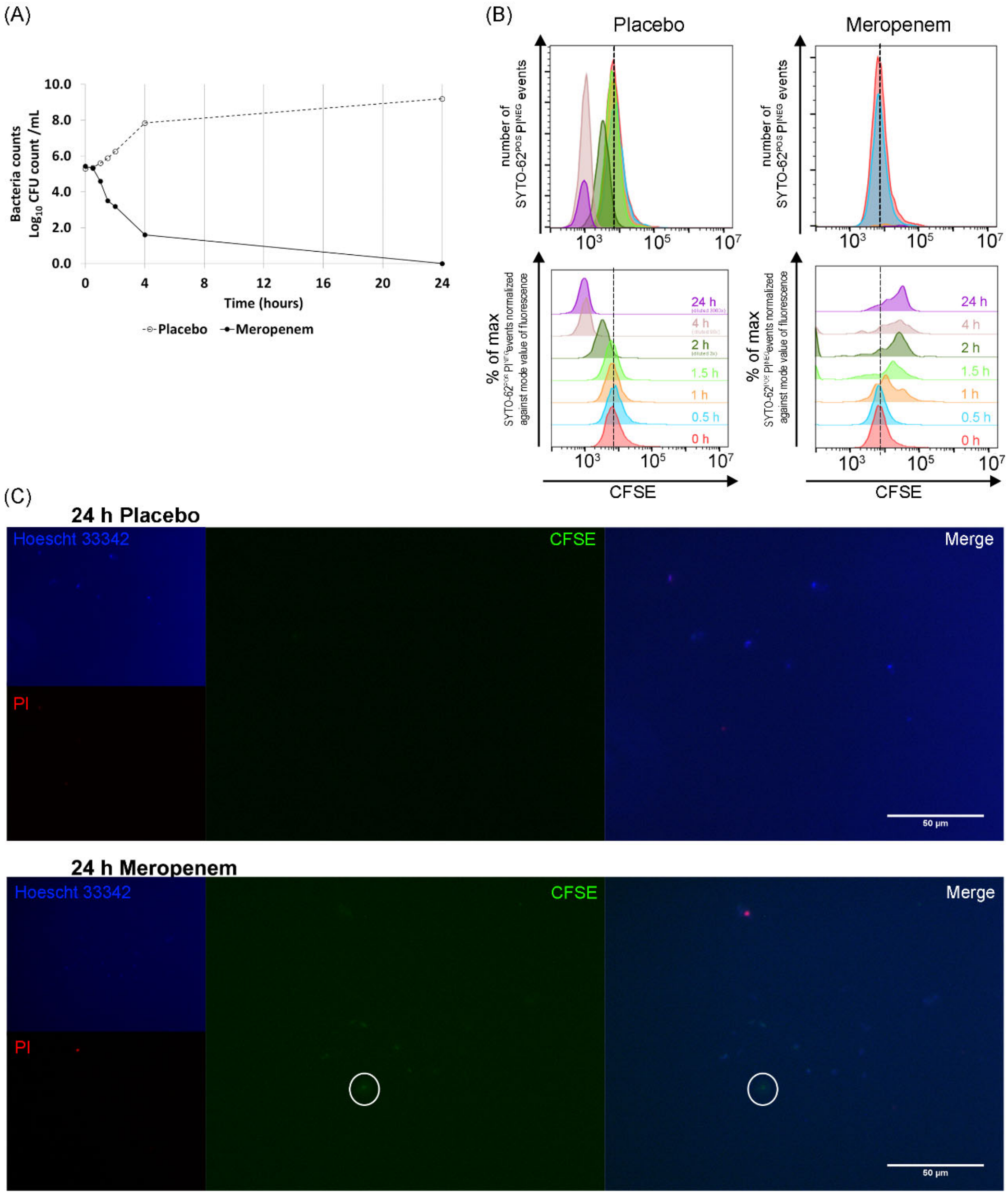

Supplementary Figure S2: Bacteria persisters manifest in clinical isolate, EC0238, after meropenem exposure

(A) Graphs showing colonies enumerated show a bi-phasic curve upon meropenem treatment, in comparison to placebo. (B) Flow cytometric histograms revealing a sub-population of bacteria surviving meropenem treatment. Dotted line marked the peak fluorescence intensity at 0 hour. (C) Widefield epi-fluorescence microscopy revealing presence of $CFSE^{High}$ bacteria, noted in white circles, when treated with meropenem at 24 hours. Scale bar represents 50 microns.

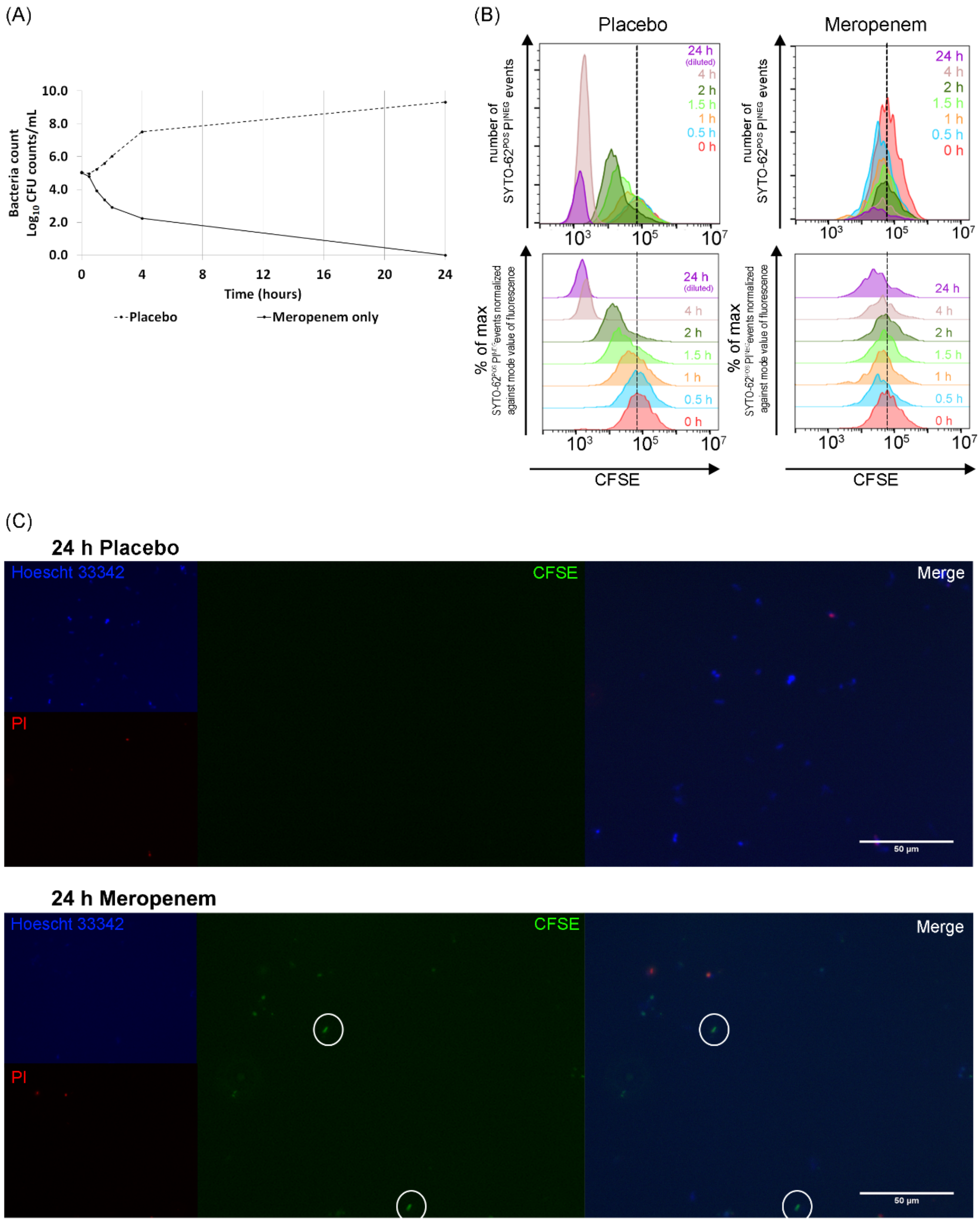

Supplementary Figure S3: Bacteria persisters manifest in clinical isolate, EC0381, after meropenem exposure

(A) Graphs showing colonies enumerated show a bi-phasic curve upon meropenem treatment, in comparison to placebo. (B) Flow cytometric histograms revealing a sub-population of bacteria surviving meropenem treatment. Dotted line marked the peak fluorescence intensity at 0 hour. (C) Widefield epi-fluorescence microscopy revealing presence of $CFSE^{High}$ bacteria, noted in white circles, when treated with meropenem at 24 hours. Scale bar represents 50 microns.

B. Details of clinical isolates manifested with persisters

Resistance genes and serotypes information were summarized in **Supplementary Table S1**. MICs performed on the isolates against the antibiotics screened were summarized in **Supplementary Table S3**.

Supplementary Table S2: Resistance gene and molecular information of the clinical isolates

**Isolates**	**EC0210**	**EC0238**	**EC0381**
Sites obtained	SSTI	Abdominal	Stool (Rectal)
Serotype (STs)	3054	69	6025
**Resistance genes**			
bla_{SHV}	-	-	ND
bla_{TEM}	+	+	ND
$bla_{CTX\text{-}M}$	-	CTX-M-3-146	-
$bla_{OXA\text{-}48}$	-	-	-
CPE	KPC-2	KPC-2	KPC-2
AmpC	-	-	ND
LEV_PABN	ND	+	ND
tetA)	+	+	-

ND = not done

Supplementary Table S3: MICs of respective antibiotics screened for clinical isolated identified to manifest with persisters

**Clinically achievable concentrations of antibiotics used to screen for persisters (mg/L)**		**MIC (mg/L) for strain**		
		**EC0210**	**EC0238**	**EC0381**
Amikacin (AMK)	65	8	64	2
Ceftazidime-Avibactam (CZA)	21/5.25	0.38/4	0.5/4	0.5/4

Levofloxacin (LVX)	8	≥ 64	1	≤ 0.25
Meropenem (MEM)	20	4	8	8
Polymyxin B (PMB)	2	0.5	2	≤ 0.25

2. Materials and methods

A. Flow cytometry

I. Flow cytometer configuration

A Cytoflex® flow cytometer (Beckman Coulter, Brea, CA, USA) at the basic 4+3+2 configuration was used in this study. The flow cytometer is equipped with 405 nm, 488 nm and 640 nm lasers. A summary of fluorophores detected by corresponding detectors and gain voltages applied to the respective detectors were shown in **Supplementary Table S4**.

Supplementary Table S4: Flow cytometry acquisition summary

Dyes/Fluorophores (final concentration used)	Fluorescence channel (excitation wavelength; emission filter used)	Gain voltages applied on the detectors
CFSE (150 μM)	FL1-H - FITC (488 nm; 525/40 filter)	800 V
Propidium iodide (20 μM)	FL3-H - PC5.5 (488 nm; 690/50 filter)	150 V
SYTO-62 (1 μM)	FL5-H - APC (640 nm; 660/10 filter)	800 V

II. Sample acquisition settings

Flow cytometric data were acquired using the complementing CytExpert software (version 2.5). A manual threshold of 3000 on both SSC-H and FSC-H parameters to remove noise. A total of 10,000 SYTO-62^{POSTIVE} events were acquired per sample. A flow rate of 50 to 150 events per second was used to assess the bacteria. Fluidics were set to acquire samples at a defaulted slow setting (10 μL/min) to reduce false positives from doublets. Samples (placebo) exceeding 350 events per second were diluted in Ca-MH broth and analysed immediately. This is to prevent overwhelming the detectors and generate false doublets. Reduction of doublets acquisition is further monitored using FSC-Height and FSC-Width parameters.

III. Data analyses

Acquired flow cytometric data were analysed using FlowJo (version 10.6; Treestar ® FlowJo, LLC, Ashland, OR, USA). Compensation against spectral overlap was also performed on the FlowJo software. Gating strategy used for data analysis were shown in **Supplementary Figure S4**. Flow cytometric data were presented as overlaid or half-staggered histograms throughout manuscript for simplicity.

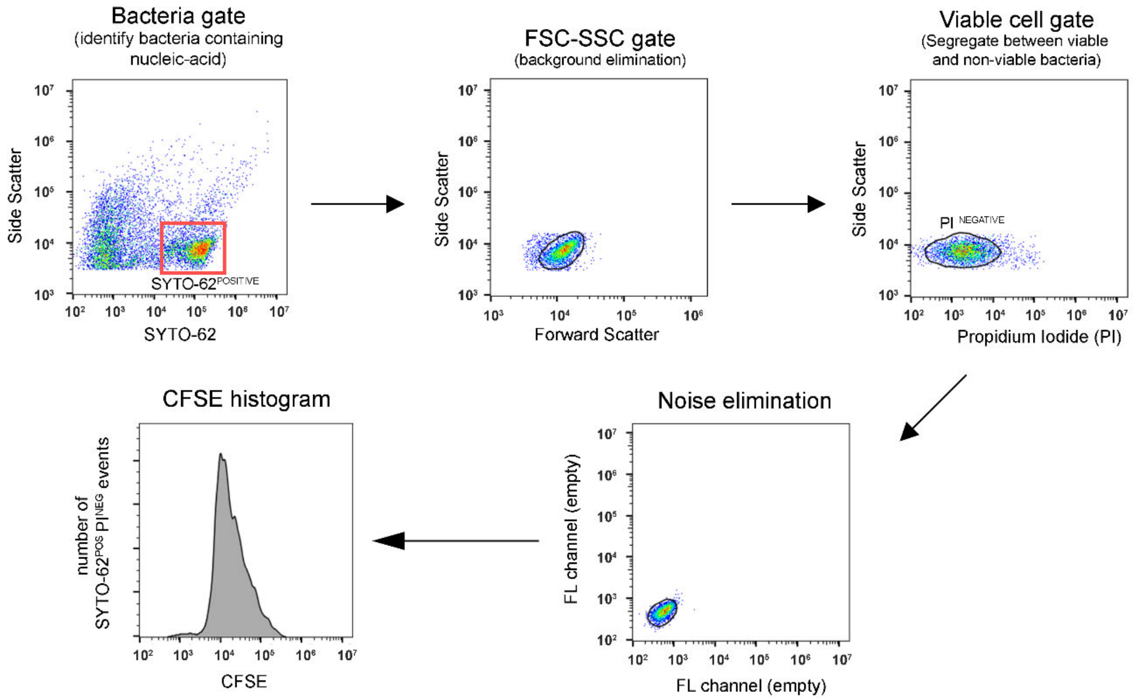

Supplementary Figure S4: Gating strategy for flow cytometry to identify persisters

Multiple gates were in place to accurately identify persisters via flow cytometry. Side scatter (determined granularity of cells) and SYTO-62 (nucleic acid-containing bacteria) were used to identify bacteria cells. A forward-scatter and side scatter (FSC-SSC) gate were to eliminate background. Viability of bacteria were determined by low/negligible uptake propidium iodide (PI). Henceforth, any viable bacteria was termed to have a phenotype of SYTO-62^{POS}PI^{NEG}. To further increase accuracy, noise elimination was done by gating discrete populations in empty fluorescence channels not in use. Viable bacteria were then assessed for CFSE fluorescence intensity (histogram). Histograms were then overlaid in a staggered manner as presented throughout the manuscript.

B. Widefield epi-fluorescence microscopy of live bacteria

I. Staining live bacteria for fluorescence microscopy

After 24 hours of respective treatments, the CFSE-labelled bacteria were spun at 3,000 rpm for 5 mins and washed with Phosphate Buffered Saline (PBS) twice. At the last PBS, pelleted bacteria were resuspended in 50 μL of PBS. Pelleted bacteria were stained with 0.2 mg/mL Hoechst 33442 at room temperature for 15 mins under dark conditions. Similar to SYTO-62 used in flow cytometry experiments, Hoechst 33342 stains nucleic acid in bacteria cells. To the Hoescht-stained bacteria, 20 μM propidium iodide were added and stained for a further 1 minute at room temperature.

Excess dyes were then washed with 1 mL of PBS and spun at 3,000 rpm for 5 mins. Supernatant containing excess dyes were carefully removed to prevent disturbance to the pellet. Pelleted bacteria were resuspended with residual PBS supernatant (< 20 μL). Bacteria suspension was then deposited onto a 10% heat-inactivated Bovine Serum Albumin-smear coated glass slide marked with PAP-pen. Glass slide was mounted with a coverslip and sealed with nail polish. Slides were imaged immediately on the microscope right after nail polish fully solidifies.

II. Fluorescence microscope configuration and image acquisition

Stained bacteria were visualised using a Nikon-Ti microscope equipped with a S-Plan Fluor ELWD 40x dry objective lens (Numerical Aperture = 0.6), Nikon Intensilight illumination source and a Nikon DS-Ri1-U3 camera. Fluorophores were excited using the Intensilight fiber. Emitted fluorescence were observed using conventional DAPI, FITC and TRITC filters equipped on the microscope. Fluorescence images was acquired using filters equipped on the microscope, which corresponded to respective fluorophores emission wavelengths. A summary of exposure time and analog gain of the detectors can be found in **Table S5**.

Micrographs acquired were exported as .tif files using the NIS-elements AR software (version 4.2). Images were further processed on ImageJ FIJI for adding of scale bar and merging images from all fluorescence channels.

Table S5: Acquisition settings on the Nikon Ti-Microscope

**Dyes**	**Exposure**	**Filter set used**	**Analog Gain**
Hoechst 33442	ME 400 milliseconds	DAPI	1.00
CFSE	ME 600 milliseconds	FITC	4.80
Propidium Iodide	ME 800 milliseconds	TRITC	2.00

3. References (for supplementary information)

1. Wong FH-S, Cai Y, Leck H, Lim T-P, Teo JQ-M, Lee W, Koh TH, Tan TT, Tan KW, Kwa AL-H. 2020. Determining the Development of Persisters in Extensively Drug-Resistant Acinetobacter baumannii upon Exposure to Polymyxin B-Based Antibiotic Combinations Using Flow Cytometry. Antimicrob Agents Chemother 64:1–15.

2. Balaban NQ, Helaine S, Lewis K, Ackermann M, Aldridge B, Andersson DI, Brynildsen MP, Bumann D, Camilli A, Collins JJ, Dehio C, Fortune S, Ghigo JM, Hardt WD, Harms A, Heinemann M, Hung DT, Jenal U, Levin BR, Michiels J, Storz G, Tan MW, Tenson T, Van Melderen L, Zinkernagel A. 2019. Definitions and guidelines for research on antibiotic persistence. Nat Rev Microbiol 17:441–448.

3. Bigger JW. 1944. Treatment of Staphylococcal Infections With Penicillin By Intermittent Sterilisation. Lancet 244:497–500.
