# Supplementary figures and images for: Ironing out Persisters? Revisiting the Iron Chelation Strategy to Target Planktonic Bacterial Persisters Harboured in Carbapenem-Resistant Escherichia coli
Source: Microorganisms. 2024 May 12;12(5):972. doi: 10.3390/microorganisms12050972 (PMC11123761; doi:10.3390/microorganisms12050972)

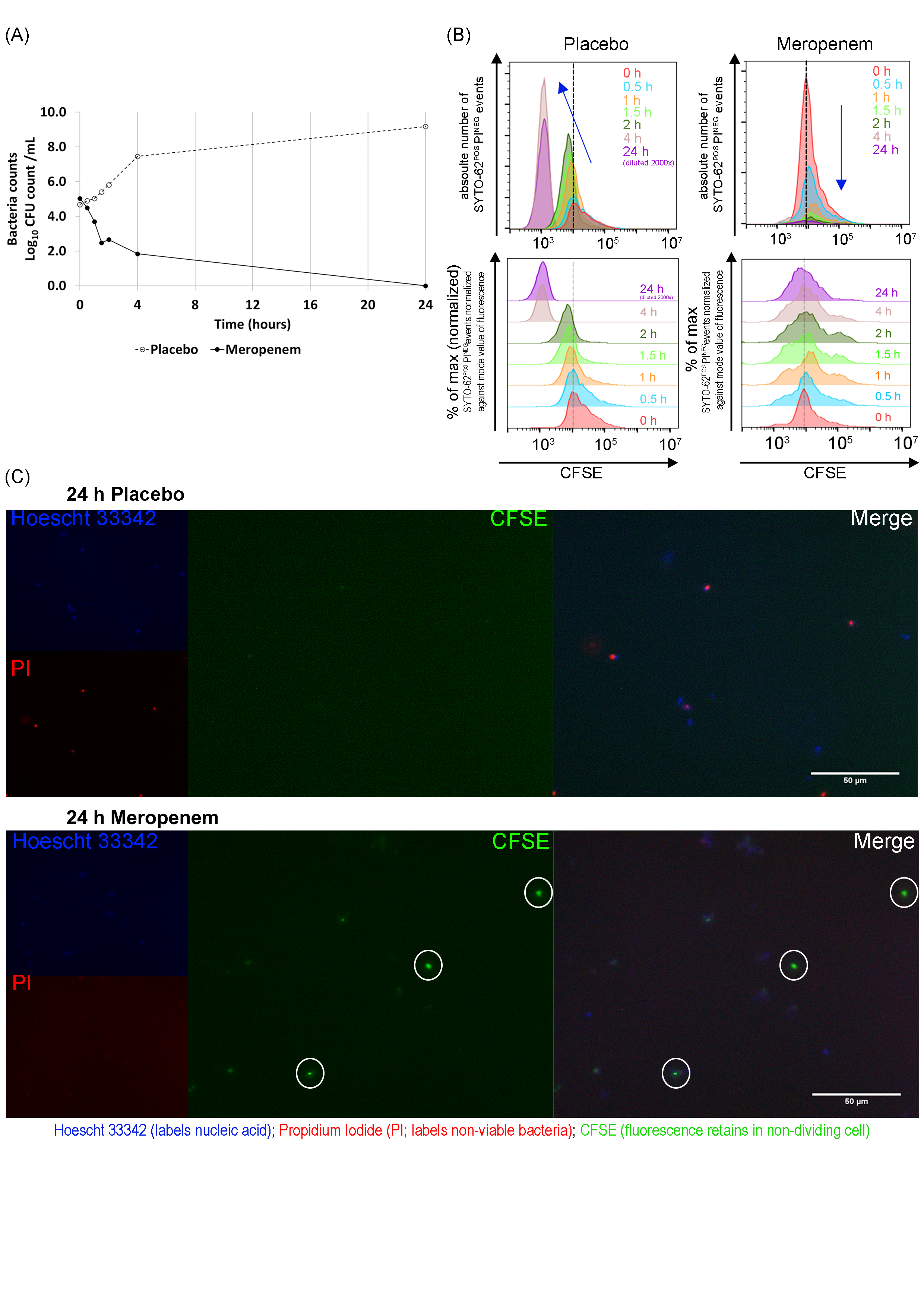

Supplement: Supplementary file 1 [file microorganisms-12-00972-s001.zip › Suppl Figure S1 - EC0210_screening.tif]

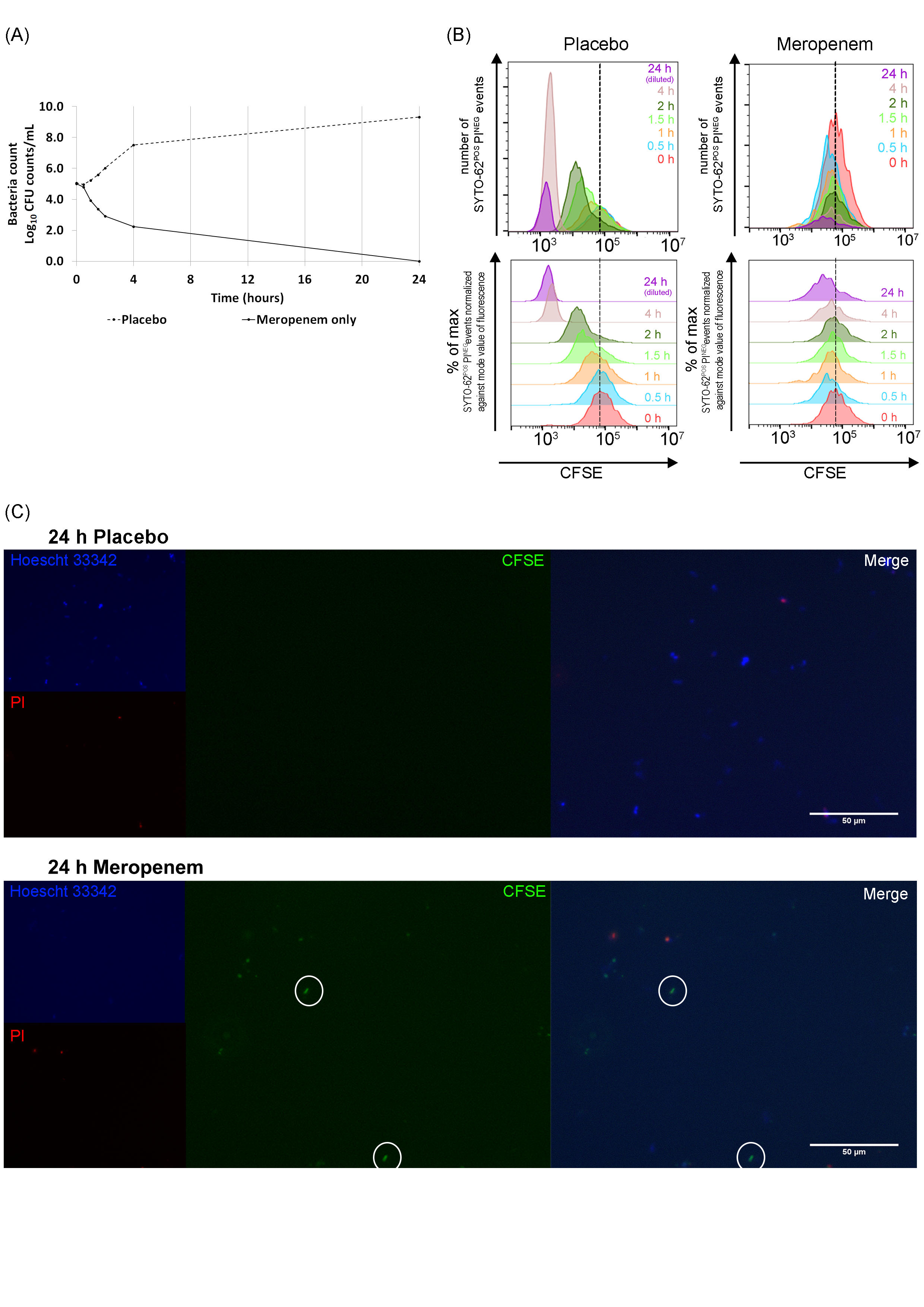

Supplement: Supplementary file 1 [file microorganisms-12-00972-s001.zip › Suppl Figure S2 - EC0381_screening summary.tif]

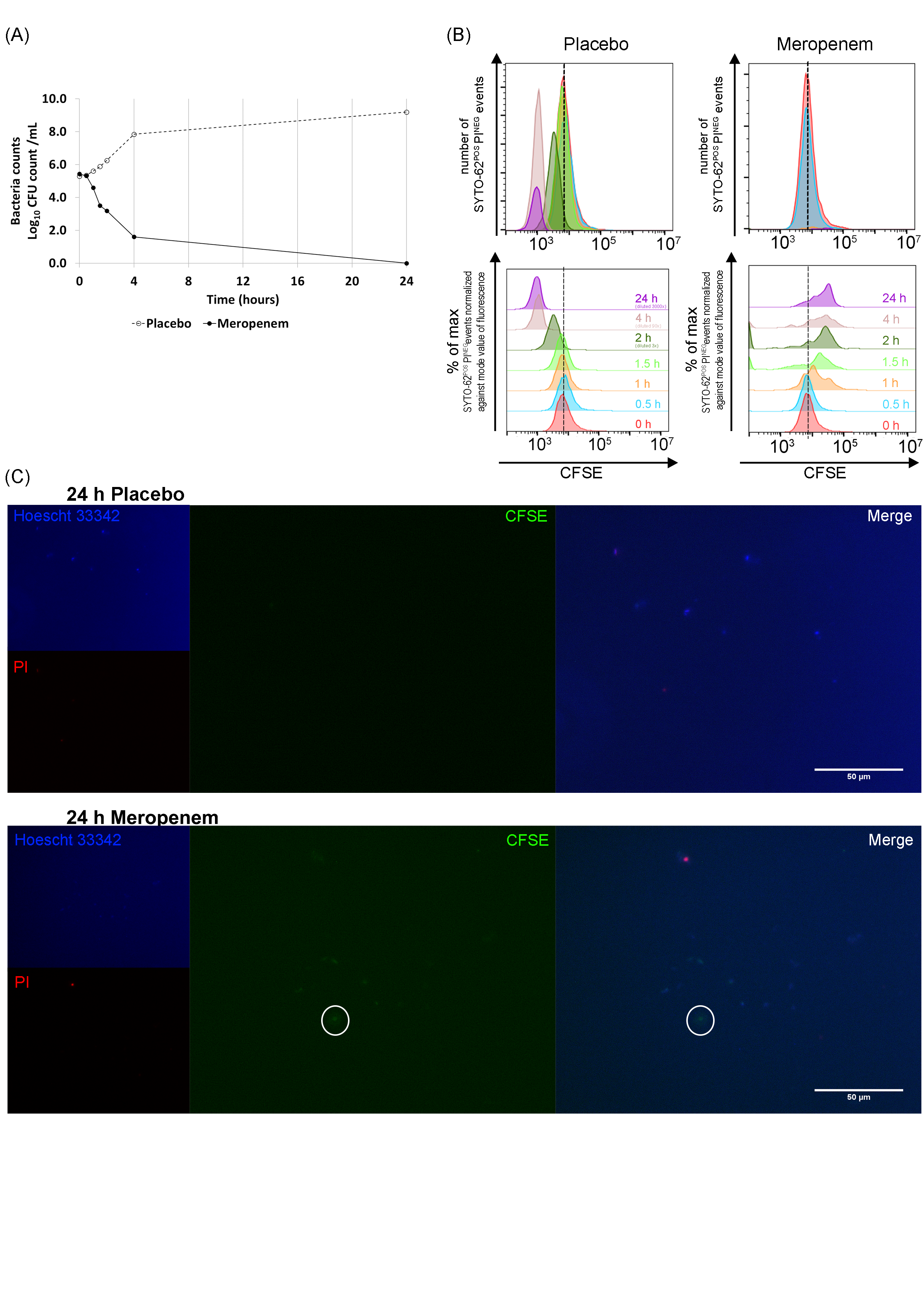

Supplement: Supplementary file 1 [file microorganisms-12-00972-s001.zip › Suppl Figure S3- EC0238_screening summary.tif]

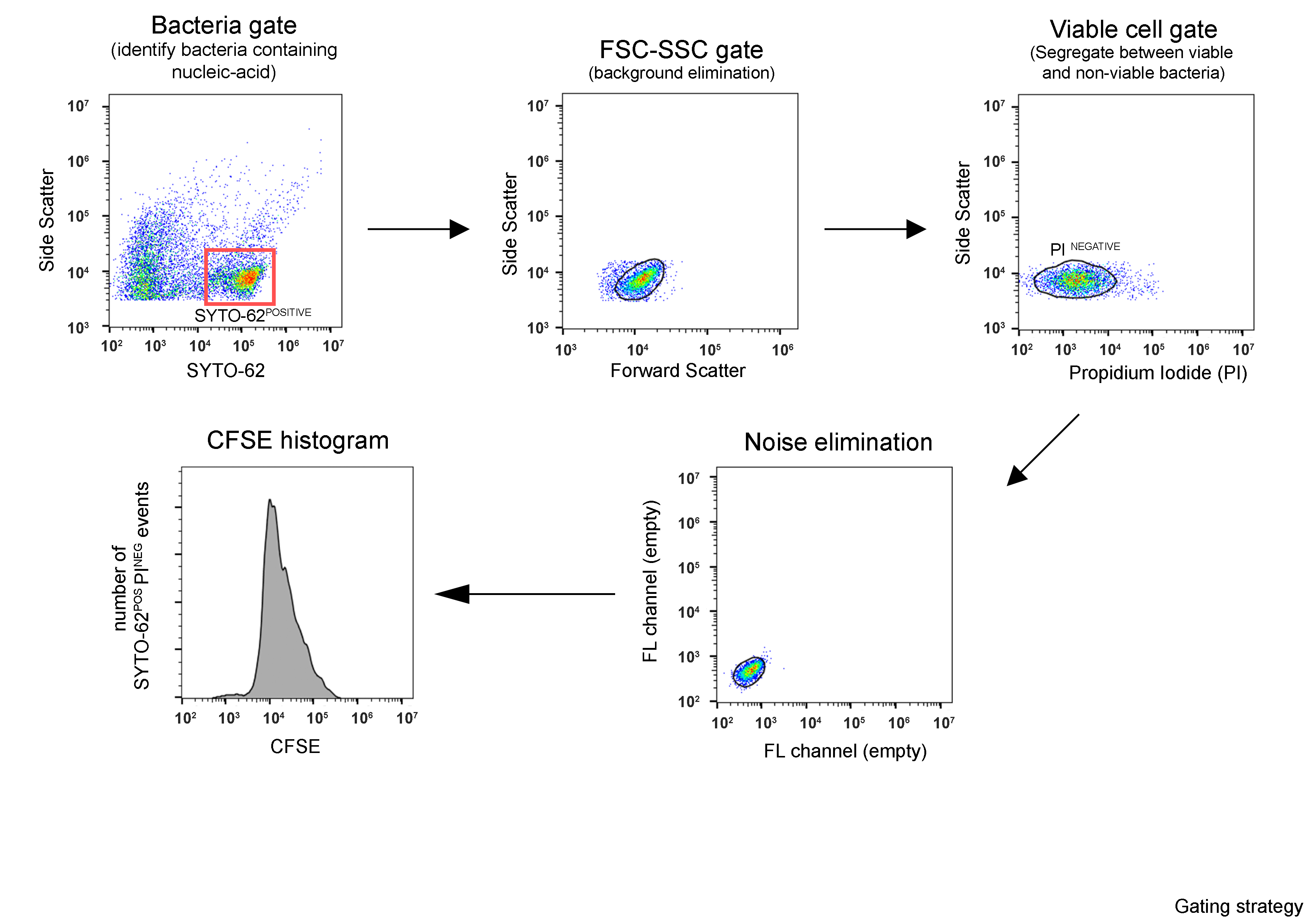

Supplement: Supplementary file 1 [file microorganisms-12-00972-s001.zip › Suppl Figure S4 - Gating Strategy.tif]
